# Endothelial Damage, Inflammation and Immunity in Chronic Kidney Disease

**DOI:** 10.3390/toxins12060361

**Published:** 2020-06-01

**Authors:** Maribel Diaz-Ricart, Sergi Torramade-Moix, Georgina Pascual, Marta Palomo, Ana Belen Moreno-Castaño, Julia Martinez-Sanchez, Manel Vera, Aleix Cases, Gines Escolar

**Affiliations:** 1 Hematopathology, Pathology Department, Center for Biomedical Diagnosis (CDB), Hospital Clinic, 08036 Barcelona, Spain; torramade@clinic.cat (S.T.-M.); mpalomo@carrerasresearch.org (M.P.); abmoreno@clinic.cat (A.B.M.-C.); jmartinez@carrerasresearch.org (J.M.-S.); gescolar@clinic.cat (G.E.); 2 Institut d’Investigacions Biomèdiques August Pi i Sunyer (IDIBAPS), Universitat de Barcelona, 08036 Barcelona, Spain; 3 Barcelona Endothelium Team, 08036 Barcelona, Spain; 4 Arrels Centre Dental, 08912 Badalona, Spain; georgina.pd@gmail.com; 5 Josep Carreras Leukaemia Research Institute, Hospital Clinic/University of Barcelona Campus, 08036 Barcelona, Spain; 6 Nephrology Department. Hospital Clinic, 08036 Barcelona, Spain; mvera@clinic.cat (M.V.); acases@clinic.cat (A.C.)

**Keywords:** chronic kidney disease (CKD), uremia, inflammation, oxidative stress, innate immunity, endothelial cells, endothelial damage

## Abstract

Chronic kidney disease (CKD) patients have an accelerated atherosclerosis, increased risk of thrombotic-ischemic complications, and excessive mortality rates when compared with the general population. There is also evidence of an endothelial damage in which the proinflammatory state, the enhanced oxidative stress, or the accumulation of toxins due to their reduced renal clearance in uremia play a role. Further, there is evidence that uremic endothelial cells are both involved in and victims of the activation of the innate immunity. Uremic endothelial cells produce danger associated molecular patterns (DAMPS), which by binding to specific pattern recognition receptors expressed in multiple cells, including endothelial cells, induce the expression of adhesion molecules, the production of proinflammatory cytokines and an enhanced production of reactive oxygen species in endothelial cells, which constitute a link between immunity and inflammation. The connection between endothelial damage, inflammation and defective immunity in uremia will be reviewed here.

## 1. Introduction

Chronic kidney disease (CKD) is a major public health issue worldwide because of its increasing prevalence, high rates of morbidity and mortality and association with poorer quality of life, reduced life expectancy, as well as high associated costs, resulting in a high burden to the healthcare systems [1,2]. CKD is associated with several complications, such as hypertension, increased cardiovascular morbidity and mortality, anemia, bone-mineral disorders, malnutrition, inflammation, sarcopenia, metabolic acidosis, and an impaired immune response, among others [3]. Cardiovascular disease is the leading cause of morbidity and mortality in patients with CKD or end-stage renal disease (ESRD) [4]. Although the prevalence of traditional cardiovascular risk factors is high in this subset of patients, it does not adequately explain the high cardiovascular burden in this population [5,6]. Thus, the role of nontraditional and/or uremia-related risk factors have been proposed to explain this enhanced risk [6], and among them the presence of an endothelial damage (ED) [7].

Endothelial cells (ECs) play a critical role in the pathophysiology of vascular disorders [8]. Endothelial activation is characterized by a dysregulation of the physiological functions of the vascular endothelium, resulting in reduced vasodilating capacity, enhanced proinflammatory and prothrombotic properties, and abnormal modulation of vascular smooth muscle cell growth and migration [7].

CKD and ESRD patients are also prone to infectious complications, because of a dysfunctional immune system, which is the second mortality cause in this population [9]. In addition, the immune system also intervenes actively in the systemic inflammation present in CKD [10,11]. In fact, among the Chronic Renal Insufficiency Cohort (CRIC) study participants, there was an inverse association between biomarkers of inflammation and measures of kidney function, such as the estimated glomerular filtration rate (eGFR) or serum cystatin C, and a positive association with the degree of albuminuria [12].

Innate immunity acts as the first defense line against infections. It is characterized by responses against specific pathogen-associated molecular patterns (PAMPs) or damage-associated molecular patterns (DAMPs), like high-mobility group box 1 (HMGB1) protein and adenosine 5’-triphosphate (ATP). In ESRD, the innate immune system, that involves monocytes, macrophages, granulocytes, and also endothelial cell activation, is activated together with a depletion of natural regulatory T-cells, resulting in systemic inflammation and an enhanced oxidative stress. These processes are connected with an adaptive immune deficiency due to the reduction of naïve and central memory T-cells and B-cells, dendritic cells, and altered functions of polymorphonuclear leukocytes and monocytes [10].

## 2. Endothelial Activation and Damage in Uremia

There is a large body of evidence of the presence of an endothelial injury in CKD, which is closely associated with the increased cardiovascular morbidity and mortality in this setting, and provides insights on the mechanisms involved.

In vivo and in vitro studies have clearly shown that there is an endothelial activation in CKD and ESRD patients, as shown by an impaired endothelium-dependent vasodilatation or increased serum levels of circulating cell adhesion molecules, such as soluble Intercellular Adhesion Molecule 1 (sICAM-1), Vascular Cell Adhesion Molecule 1, (sVCAM-1), and sE-selectin [13,14,15,16]; and other endothelium-derived proteins, such as monocyte chemoattractant protein-1 (MCP-1) [17], angiopoetin-2 [18], tissue factor (TF) [19,20,21], and total and active von Willebrand factor (VWF) [22,23] in the serum of these patients. It must be emphasized that endothelial activation is considered an early trigger for the development and the progression of atherosclerosis. It has been related to the accelerated atherosclerosis and the prothrombotic state present in CKD, which may explain the increased cardiovascular risk in this population, beyond traditional cardiovascular risk factors [24]. Further, markers of ED in CKD have been associated with the arterial stiffness [25], IL-8 driven vascular calcification [26] or the reduced microcirculation present in this population [27], which also contributes to the cardiovascular burden, as well as with the abnormal left ventricular structure and function [28,29,30] and, what is clinically relevant, to the increased mortality risk in CKD and ESRD [31,32,33,34,35].

**Figure 1:**
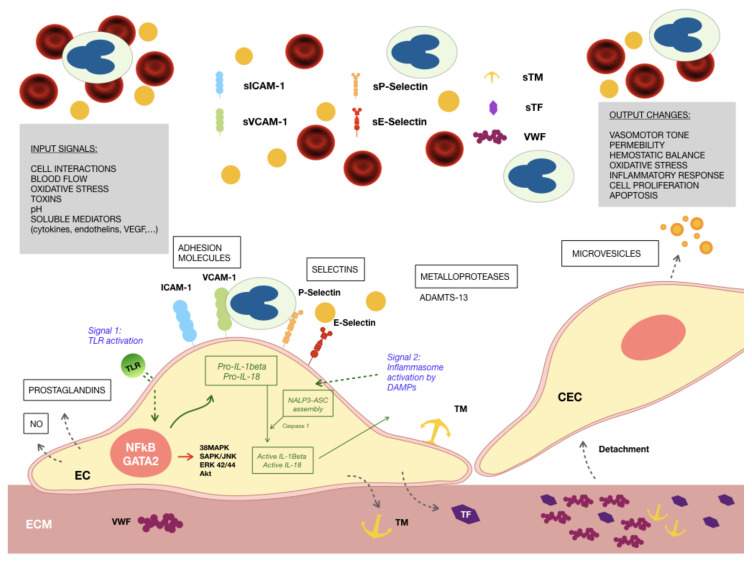
Endothelial cells exposed to the uremic environment. Different factors alter the phenotype of endothelial cells (EC), showing signs of inflammation, oxidative stress and a prothrombotic behavior of the produced extracellular matrix (ECM): increased expression of adhesion molecules, such as Intercellular Adhersion Molecule 1 (ICAM-1), Vascular Cell Adhesion Molecule 1 (VCAM-1), and P- and E- selectins, both membrane-bound and soluble; thrombogenic proteins, such as von Willebrand Factor (VWF), tissue factor (TF) and thrombomodulin (TM) secreted to the ECM; production of reactive oxygen species (ROS) intracellularly. Activation of innate immunity mechanisms occurs in response to the presence of intracellular and soluble damage-associated molecular patterns (DAMPS). Signal 1: Toll-like receptor 4 (TLR4) is overexpressed in ECs exposed to the uremic milieu, being able to detect DAMPS leading to the production of the inactive forms of interleukins 1β and 18 (IL-1β and IL-18); Signal 2: DAMPS also promote the engagement of the inflammasome NOD-like receptor prying domain-containing-3 (NALP3), with the activation of IL-1β and IL-18. Activated nuclear factor kappa B (NFκB), linked to the inflammatory and oxidative stress responses of ECs to uremic media is related to increases in the degree of phosphorylation of ERK 42/44, SAPK/JNK, and AKT. These phenotypic alterations of ECs result in the recruitment of circulating leucocytes to the endothelial surface and, along with disrupted cell–cell contacts, their extravasation to the subendothelium to maintain the inflammatory response. Endothelial cells have a tendency to detach from their vascular bed passing to the circulation as circulating endothelial cells (CECs), and exposing an ECM highly reactive to circulating platelets.

The endothelium in CKD patients suffers from a continuous insult causing its activation and injury that may end in a dysfunctional state (Figure 1). The endothelial activation in CKD is attributed to the pulsatile blood flow and disturbed shear stress [36], accumulation of uremic toxins, such as dimethyl arginines [37,38], indoxyl sulfate (IS) [39,40,41], indole-3 acetic acid (IAA) [21,42], kynurenine [20,43,44], p-cresol [45,46], trimethylamine-N-oxide (TMAO) [41,47], oxidized low-density lipoprotein (LDL) cholesterol particles [48], carbamylated lipoproteins [49,50], reactive oxygen species (ROS) [39,51,52], advanced glycation end-products (AGEs) [53,54], hyperhomocysteinemia [55,56], hyperphosphatemia [57,58], bacterial lipopolysaccharides or other bacterial products [59,60,61], endogenous damage-associated molecules, and proinflammatory cytokines [62,63,64,65,66], which all together constitute the uremic environment.

Our group has thoroughly studied the endothelial damage induced by the uremic environment in an in vitro model. Cultured endothelial cells exposed to growth media containing uremic serum from patients on maintenance hemodialysis showed morphological alterations, with irregular shape and heterogeneous size, abundant presence of vacuoles, and an increased number of mitotic cells [67]. They exhibited increased proliferation, evidenced by morphological analysis, cell cycle evaluation by flow cytometry, and activation of the mitogen-activated protein kinase (MAPK) 42/44 [67]. Cells grown under uremic conditions showed inflammation signs, as demonstrated by enhanced expression of VCAM-1, ICAM-1, and endothelial-leukocyte adhesion molecule (ELAM-1) on the cell surface, as well as a higher presence of these molecules in their soluble form and activation of the protein p38MAPK [68]. No evidence of enhanced apoptosis was detected despite the accelerated proliferation observed in ECs cultures in response to the uremic media [67]. Moreover, these cells produced an extracellular matrix (ECM) characterized by a less intricate network of fibrils [19] and an increased thrombogenicity, with an enhanced expression of tissue factor (TF) [19], VWF [68], and thrombomodulin [68]; while maintaining normal activity of metalloprotease ADAMTS13 [69].

The chronic inflammatory state described concurs with an enhanced oxidative stress in CKD patients. Oxidative stress occurs as a result of the imbalance between an increased production of ROS and the limited defense capability of the natural antioxidants, both occurring in CKD. The enhanced oxidative stress in CKD could be, at least in part, responsible for the cytotoxic injury to which the endothelium of these patients is exposed. Therefore, antioxidant therapy, such as the use of compounds that potentiate antioxidant enzymes, has been proposed as an effective strategy to prevent the development of endothelial damage and hence may have the potential to reduce the cardiovascular risk in these patients [52,70].

Differential proteomic analysis of endothelial cells grown under uremic conditions [71] revealed increases in the expression of proinflammatory proteins, such as two components of the proteasome, the protein HMGB1, and the enzyme aldose reductase. In addition to these proteins, a higher expression of antioxidant enzymes, such as glutathione peroxidase, superoxide dismutase, and peroxiredoxin, was also detected, suggesting an adaptive response to the oxidative stress induced by uremic media. Interestingly, some of these proteins are directly or indirectly related to nuclear factor kappa B (NFκB), which was also overexpressed in uremic endothelial cells [69]. This transcription factor plays a crucial role in the development of inflammatory and immunological responses and oxidative stress. Furthermore, there is evidence of an activation of the innate immunity in uremic endothelial cells, as demonstrated by an enhanced expression of Toll-like receptor 4 (TLR4) on their surface and the activation of the inflammasome nucleotide-binding oligomerization domain (NOD)-like receptor prying domain-containing-3 (NLRP3, also known as NALP3) [65].

Other experimental studies have shown enhanced apoptosis [72,73], increased expression of proinflammatory proteins [16], augmented production of intracellular ROS [39], enhanced cell senescence [74], and a higher exposure of phosphatidylserine, which may contribute to the prothrombotic state in CKD [75]. In a recent study, uremic sera increased the levels of miR-92a in cultured endothelial cells and suppressed the expression of miR-92a targets, such as sirtuin 1 (SIRT1), Krüppel-like factor 2 (KLF2), and KLF4, three endothelial-protective molecules, as well as the expression of endothelial nitric oxide synthase (eNOS). In addition, there was an increase in caspase 1, a hallmark of inflammasome activation [70]. Uremic medium also impairs the endothelial barrier function and repairs capacity by disrupting cell–cell contacts, associated with a decreased expression of vascular endothelial (VE)-cadherin and zonula occludens 1 (ZO1) [76,77].

The transcription factor aryl hydrocarbon receptor (AHR) also plays a crucial role in the development of endothelial damage in CKD. The accumulation of toxins derived from tryptophan metabolism, such as IS, IAA, and kynurenine, in CKD cause the activation of AHR in different cells and especially in endothelial cells [20]. In CKD, endothelial AHR activation has a prothrombotic action by promoting the production of TF by non-genomic pathways [21]. IAA, acting through AHR, has been reported to activate p38MAPK, with the induction of NFκB activation, which is the element that binds to the promoter (F3) of TF [78]. Interestingly, AHR can be also activated by laminar shear stress [79], exhibiting an atheroprotective action. In this regard, Lano et al. [80] provided evidence indicating that the antithrombotic properties of shear stress on the endothelium could be impaired by toxins, such as IS, that act as agonists for AHR and, therefore, contribute to the cardiovascular risk in CKD.

Circulating endothelial cells (CECs) are mature endothelial cells present in blood and are considered a marker of an ongoing endothelial injury. Increased CEC values have been described in CKD patients [69]. In addition, higher CEC counts in patients in hemodialysis were directly associated with a higher incidence of cardiovascular events [81,82]. Endothelial progenitor cells (EPCs), which are originated in the bone marrow and circulate in blood, are thought to be involved in the damaged endothelium repair. Circulating EPC numbers and functionality are decreased in patients with CKD or dialysis [8,83,84,85], reflecting a reduced ability to restore the damaged endothelium, and have been shown to be predictors of future adverse outcomes in this population [86,87,88]. In addition, microvesicles (MV) are extracellular vesicles released from the plasma membrane of activated or apoptotic cells. There is increasing evidence of a role for MV in intercellular communication in different processes, especially in vascular biology and also in hemostasis [89]. Endothelial MV (EMV) intervene in pathophysiological processes by interacting and fusing to target cells, such as leukocytes, releasing their contents directly into their cytoplasm and modifying their biological behavior. EMV have been found to be increased in CKD or dialysis patients [53,75,90,91] and have been associated with vascular damage [53] and cardiovascular mortality [90,91,92].

## 3. The Endothelium, Inflammation and Immunity

The inflammation associated with CKD is in part due to the activation of the elements that participate in the innate immune system, including monocytes, macrophages, granulocytes, and other cellular types of the body. The immune deficiency present in CKD is mainly caused by a reduction of antigen-presenting dendritic cells, T- and B-cells and alterations in the phagocytic capabilities of monocytes and polymorphonuclear leukocytes (PMNs) [10]. In advanced CKD, there is evidence of a senescence-associated secretory phenotype, characterized by a defective regulation of inflammatory processes with release of cytokines from uremic senescent cells [93].

**Figure 2:**
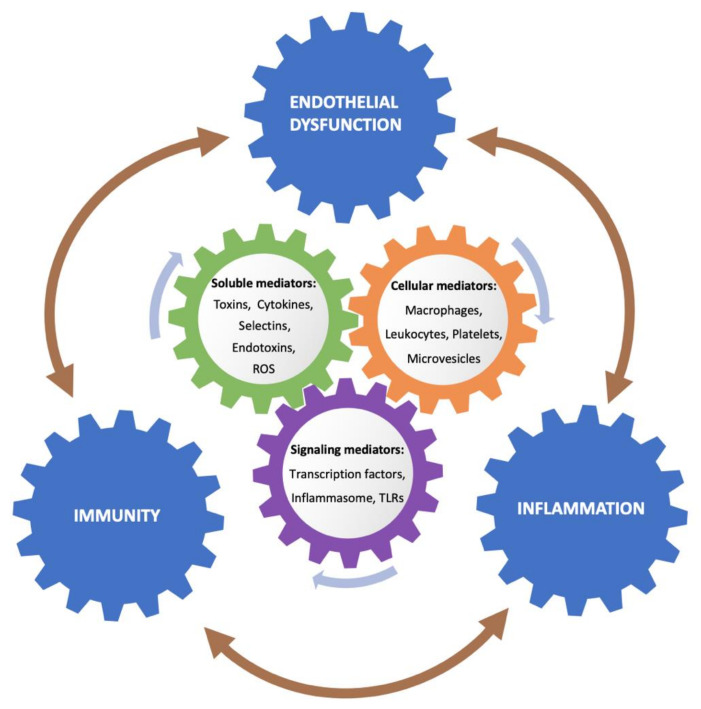
The mechanisms summarized inside the cycle do not correspond exclusively to the interaction between two processes, but may be implied to different extent in development of other alterations. Exposure of the endothelium to the uremic milieu leads to a cross-talk between inflammation, immunity and endothelial activation through several mediators. The uremic media consists of soluble factors secreted by cells and tissues to the circulation, such as cytokines, adhesion receptors, coagulation proteins, and selectins; products are derived from the uremic state or induced by renal replacement therapy (RRT), such as endotoxins and toxins, and from an increased oxidative stress, such as reactive oxygen species (ROS). Cellular response involves macrophages and leukocytes, and to a lesser extent platelets and circulating microvesicles secreted from injured cells. In this orchestrated response, signaling mediators play a role, with activation of transcription factors, engagement of inflammasome NALP3, and Toll-like receptor 4 (TLR4) overexpression, promoting further activation of proinflammatory mediators.

The endothelium also participates actively in innate and adaptive immune responses, aside from the basal functions in hemostasis [94]. Endothelial cells take part in blood supply, nutrient delivery, metabolism, and immune cell trafficking, among other functions. Moreover, due to their strategic location, endothelial cells are the first to detect pathogens and endogenous DAMPS in the circulation. Due to their plasticity, endothelial cells are dynamic enough to not only adapt but also respond to extracellular environmental changes that exhibit a role in the immune system. Therefore, endothelial cells play an active role not only in coagulation and inflammation, but also in innate and adaptive immunity (Figure 2).

Production of proinflammatory cytokines and chemokines by endothelial cells causes the amplification of the immune response by attracting and mediating the extravasation of immune cells. Also, endothelial cells stimulate cytokine production in this cell type. Moreover, endothelial cells can act as antigen presenting cells under certain conditions. In this regard, major histocompatibility complex (I and II) molecules expressed at the endothelial cells surface facilitates their recognition by T-cells and their tissue infiltration [95]. They express an array of accessory molecules, such as CD80, CD86, CD40, and CD134L, among others [95]. In addition, endothelial cells have a role in adaptive immunity by interacting with leukocytes [96] and also with platelets, a process in which endothelial P-selectin plays a major role [97]. Platelet adherence onto activated endothelial cells triggers inflammation [98].

The inflammation on endothelial cells in CKD is often induced by DAMPs, released by damaged cells, and toxins. Different cell types express Toll-like receptors (TLRs) and components of the inflammasome. These are elements of the innate immune response that cause microinflammation and vascular damage [99].

## 4. Inflammasomes, TLRs, Endothelium and Chronic Kidney Disease

Inflammasomes act as a connection between immunity and inflammation. Inflammasomes are multiprotein complexes that become activated in response to microbial and non-microbial proinflammatory triggers, and are assembled by pattern recognition receptors (PRRs). PRRs and inflammasomes are key components of the innate immune system for host defense. PRRs recognize PAMPs, such as nucleic acids or components of the cell wall from pathogens, and also host-derived DAMPs, released as a result of damaged cells and tissues, such as ATP and double-stranded DNA, among others. Both PAMPs and DAMPs induce proinflammatory cytokines production through the regulation of the enzymatic activity of caspases. Fatty acids, products downstream of elevated glucose levels, crystal formation, such as those induced by calcium oxalate, cholesterol emboli, and uric acid, among others, constitute DAMPs that can lead to the activation of inflammasomes.

The best characterized inflammasome is NALP3, also known as NLRP3 and cryopyrin. It consists of a sensor molecule (the intracellular receptor protein NALP3), connected to an adapter protein called apoptosis-associated speck-like protein containing a caspase activation and recruitment domain (ASC), and the inflammatory enzyme caspase-1 [100]. The assembly of the inflammasome elements induces the activation of inflammation signaling networks [101,102], including those dependent on NFκB and protein p38MAPK [103]. Oxidative stress also promotes the activation of the inflammasome through the protein thioredoxin-interacting protein (TXNIP) [102]. As a result of the inflammasome engagement, there is cleavage of precursor forms of the cytokines IL-1β and IL-18, and also of gasdermin D (GSDMD) leading to pyroptosis [104]. There are many PAMPs and DAMPs that activate the inflammasome and, therefore, these stimuli probably converge on common mechanisms of NALP3 activation. Cytosolic K+ efflux [105,106,107], mitochondrial ROS [108,109], oxidized mitochondrial DNA leakage [110], and ion fluxes because of lysosomal disruption [105,111] are some of these mechanisms.

The NALP3 inflammasome constitutes an alarm for the immune system to combat insults. However, if constantly activated, it may contribute to pathological injury itself [112]. NALP3 components are expressed in ECs [113], vascular smooth muscle cells (VSMCs) [114], and immune cells, especially phagocytic antigen presenting cells, such as macrophages and dendritic cells, and changes in their expression are associated with vascular inflammation [115]. NALP3 is highly expressed in the aorta of patients with coronary atherosclerosis and levels correlate with the stenosis severity [116]. NALP3 inflammasome contributes to atherogenesis at different stages, with IL-1β being a key product. Its production induces the expression of other proinflammatory cytokines (IL-6 and tumor necrosis factor alpha; TNF-α) [117,118]; IL-8, attracting neutrophils; monocyte chemo-attractant protein-1 (MCP-1), promoting the adhesion of circulating monocytes [119]; and VCAM-1 on endothelial cells [120], which mediates adhesion and infiltration of monocytes. Proliferation and migration of VSMCs [121,122,123] also occurs, with an increased migration ability and susceptibility of macrophages to lipid deposition accelerating foam cell formation [124], and also the expression of matrix metalloproteinases (MMPs) [125,126] promoting collagen degradation. All these events lead to plaque instability [127].

Inflammasome assembly has been involved in a number of kidney diseases, including acute kidney injury, CKD, and diabetic kidney disease, via canonical and non-canonical mechanisms that participate in the regulation of processes such as inflammation, pyroptosis, apoptosis, and fibrosis [128]. Among them, there is evidence of NALP3 as a key pathogenic mechanism of CKD [129,130,131]. NALP3 is expressed in tubular epithelial cells, glomeruli, podocytes, mesangial, and intercalated cells [128,132,133]. There are a number of primary and systemic diseases involving the kidney, most of them acute inflammatory diseases, associated with the activation of the NALP3 inflammasome [64]. Mitochondrial ROS-mediated NALP3 inflammasome activation contributes to the renal tubular cells injury caused by aldosterone [134]. IL-1β and IL-18 promote kidney injury through inflammatory cell recruitment. These proinflammatory cytokines also play a role in adaptive immunity, since they influence Th17 and Th1 responses, including CD4+ T-cells differentiation, key mediators in the pathogenesis of a number of autoimmune diseases, also in the kidney [128]. In addition, there are increased levels of caspase 1 and NALP3 inflammasome in lupus nephritis, which are associated with neutrophil extracellular traps (NET)-mediated activation in macrophages [135]. NETosis is also an activator of inflammasome engagement. 

NALP3 inflammasome is also activated in immunocompetent peripheral cell lines isolated from uremic patients undergoing dialysis treatment. These cells show higher mRNA levels of NALP3, Caspase-1 (CASP-1), ASC, IL-1β, IL-18, and P2X7 receptors compared to cells from healthy subjects [136]. Moreover, it is activated in endothelial cells in culture exposed to serum samples from patients with CKD under conservative treatment (CKD stages 4–5) and under maintenance hemodialysis or peritoneal dialysis. Interestingly, NALP3 inflammasome engagement was more notable in association with the serum samples from patients under RRT, especially peritoneal dialysis [65]. Of note, patients included in this study were carefully selected excluding other cardiovascular risk factors in order to evaluate the effect of uremia per se. Diabetes is also a condition in which inflammasomes seem to be activated, although it is unclear whether hyperglycemia has a direct effect. In patients with diabetic kidney disease, IL-1β and IL-18 levels are elevated [128,133].

Therefore, there is increasing evidence of the role of NALP3 inflammasome in the development of a number of renal diseases and related complications. Although, the exact mechanisms remained to be deciphered, it may constitute a therapeutic target once better known.

One of the key functions of innate immunity is the recognition of PAMPs and DAMPs through PRRs, which are receptors for cell stress and damage signals. Most TLRs are a type of transmembrane PRRs. They recognize various common pathogenic components, such as viral RNA, bacterial oligodeoxynucleotides, lipopolysaccharides (LPSs), and peptidoglycans, among others. TLRs trigger signals that result in the expression of proinflammatory genes, leukocyte chemotaxis, cytotoxicity, phagocytosis, and induction of the adaptive immune responses [137,138]. They have an initial protective role, but when they receive stimuli intense enough and persistent over time their activation may lead to a pathological inflammatory response.

TLRs are also related to inflammasomes, since their engagement occurs after an initial step in which TLRs activation triggers NFκB dependent gene transcription of pro-IL-1β and pro-IL-18. These proteins are released to the cytoplasm but require cleavage by caspases, produced by the assembly of NALP3 in a second step, to become activated and secreted [128] (Figure 1).

TLRs stimulation leads to the release of inflammation mediators, such as IL-6, IL-8, and TNF-α, among others. In relation to this, the uremic environment causes changes in the activation of TLRs, although the tendency of these changes seems to be controversial or at least dependent on the cell type. As reviewed by Kato et al. [11], uremia diminishes the capabilities of dendritic cells and macrophages for antigen presentation with alterations in costimulatory molecules (CD80, CD86) [139], and the expression of these molecules is regulated by TLRs. In some studies, TLR4 expression has been shown to be constitutively decreased in monocytes in both predialysis ESRD [140] and hemodialysis (HD) patients, in which endotoxins contained in the dialysate or derived from the gut microbiota may also contribute [141]. On the other hand, other studies provide evidence demonstrating that the uremic environment induces enhanced expression of TLR4, together with cytokine production, thus promoting inflammation [142]. In this regard, TLR activation has been related to the inflammatory and profibrotic effects of prolonged exposure to peritoneal dialysis solutions, either conventional or more biocompatible. Anti-TLR strategies, such as sTLR2, were proposed to inhibit peritoneal infection-induced fibrosis without compromising bacterial clearance and also as an antifibrotic strategy [143]. Moreover, TLR4 upregulation with activation of inflammatory signals, some dependent on TNF-α and NFκB, has been also described to be involved in the muscle inflammation associated with CKD [144]. Therefore, it seems that there is a dysregulation of the expression of TLRs in uremia.

Different TLRs, including TLR4, are constitutively expressed in endothelial cells [65,94]. TLR4 increases its expression on the endothelial cell surface under inflammatory conditions. A wide range of DAMPs released after cellular stress, such as HMGB-1, which is upregulated in endothelial cells exposed to uremic media [71], can activate TLRs, especially TLR2 and TLR4, causing the induction and amplification of the inflammatory response [137] through the transcription factor NFκB activation. Stimulation of TLR4 by HMBG1 in response to the uremic toxin TMAO results in an increased permeability of vascular endothelium due to the disruption of cell to cell junctions [145,146]. Intravenous injection of high-density lipoprotein (HDL) cholesterol from patients with CKD into mice increased blood pressure, an effect mediated by TLR2 expressed on the endothelial cells surface [147]. In vitro exposure of endothelial cells to the uremic milieu causes upregulation of TLR4 with its increased expression on the cell membrane, together with an augmented generation of intracellular ROS [65]. These changes are associated with an elevation of adhesion receptors at the cell surface, with activation of the intracellular cell-stress related signaling protein AKT and the transcription factor NFκB. Interestingly, blockade of TLR4 partially prevented these effects as well as the activation of the protein TXNIP, an element of the NALP3 inflammasome [65].

There is evidence of soluble extracellular TLR4 (sTLR4), which has shown the capability of diminishing TLR4 signaling. sTLR4 levels are increased in HD patients with subclinical inflammation, but not in non-inflamed HD patients, vs. healthy control subjects. Therefore, sTLR4 release could be a key counter-regulatory mechanism to modulate inflammation in this setting [148,149].

## 5. Gut Dysbiosis in CKD and Uremic Toxins: Role in Inflammation, Oxidative Stress and Endothelial Activation

The healthy gut microbiome has a symbiotic relationship with the host, helping in the digestion of dietary fiber, the generation of beneficial short-chain fatty acids (SCFA), the synthesis of vitamins and amino acids, maintaining the intestinal barrier function, and modulating the immune system and metabolism [150]. Gut dysbiosis has been linked to several diseases, such as inflammatory bowel disease, obesity, type 2 diabetes mellitus, cancer, cardiovascular disease, or CKD [150]. Changes in the gastrointestinal tract (GI) and intestinal barrier function and in the composition of the gut microbiota have been related to many complications of CKD and ESRD [151,152].

There is an impaired function of the intestinal barrier in uremia [152], with an increased intestinal permeability to different size macromolecules, as observed in animal models and CKD patients [153]. The impaired renal function elicits an increase of urea and uric acid levels in the GI tract in CKD and ESRD. Urea is metabolized by urease-containing bacteria to ammonia, which is associated with an increase in the GI pH and a decrease in the tight junctions in the GI tract epithelial cells [154,155], thus increasing GI permeability, allowing the translocation of bacteria or bacterial products to the systemic circulation [152]. There is evidence of elevated levels of endotoxins in patients with advanced CKD [156,157,158,159], which are associated with increased inflammation and activation of the immune system [151,152,155,159]. A very recent study [160] questioned the enhanced gut derived bacterial products translocation in a selected group of CKD and dialysis patients without metabolic or inflammatory disease.

CKD is related to gut dysbiosis, due to the uremic condition, dietary restrictions, administered drugs (antibiotics, phosphate binders, oral iron supplementation), and hypervolemia, with associated intestinal wall congestion and edema [151,155]. Altogether these conditions induce a significant loss of the gut microbiota diversity, with a reduction in commensal SCFA-producing bacteria and an expansion of bacteria that contain urease, uricase, and indole and p-cresol-forming enzymes. This change, from a sacharolytic to a proteolytic fermentation microbiota, in CKD leads to a reduced formation of SCFA and an increased production of uremic toxins, such as IS, p-Cresyl sulfate (PCS), IAA, ammonia, phenylacetylglutamine, or TMAO, among others [161,162,163,164]. As glomerular filtration is reduced, uremic toxins progressively accumulate. Further, IS and PCS are highly bound to proteins, limiting the efficacy of dialysis for their elimination. Accumulation of uremic toxins is involved in oxidative stress, systemic inflammation, CKD progression, and a higher cardiovascular risk and mortality, as well as other CKD-related complications, [151,155,164].

The gut-derived toxins previously mentioned have a deleterious impact on the endothelium. IS has been associated with increased CV morbidity and mortality in CKD patients [165]. IS increases the formation of endothelial microvesicles (EMV), decreases nitric oxide (NO) bioavailability, promotes production of ROS, induces the expression of adhesion molecules via the NFκB pathway, increases endothelial permeability by decreasing VE-cadherin expression, reduces EPCs mobilization and angiogenesis, and accelerates EPCs senescence. It also increases the expression and procoagulant activity of endothelial cell TF, and has been associated with immune-mediated endothelial damage [166]. PCS also exhibits a role in endothelial activation, through increased expression of ICAM-1, MCP-1 and TF, which promotes adhesion of leukocytes to the endothelium. It inhibits proliferation, viability, and repair capabilities impairing nitric oxide (NO) signaling or increasing its permeability [167]. TMAO, a gut metabolite derived from the fermentation of dietary choline/phosphatidylcholine, L-carnitine or betaine, also induces endothelial damage, with activation of NALP3 inflammasome [168], cellular inflammation, ROS production, and has been associated with atherothrombosis [169].

## 6. Uremia, Platelet Dysfunction and Alterations in Immunity

Alterations of platelet functions have been widely recognized in patients with CKD. Paradoxically, a bleeding tendency coexists with accelerated atherosclerosis and an enhanced risk of thrombosis in these patients (see [170] for extended review). The inflammatory state in CKD patients has been associated with a pre-existent platelet dysfunction [171]. In addition, platelet microparticles (PMPs) are increased in CKD and dialysis patients [91] and have been reported to express 50- to 100-fold more procoagulant capacity than activated intact platelets, with the ability to activate the classical complement pathway. The microRNAs carried by vesicles from platelets, endothelial cells, and monocytes have potential inflammatory effects [172]. Further, the gut dysbiosis in CKD may play a role in the thromboembolic complication in CKD. IS promotes platelet hyperactivity [173] and the binding of bacterial derived lipopolysaccharides to endothelial cells and platelets through TLRs, while TMAO also causes platelet hyperactivity that may promote thrombus formation [174].

Platelets have functions beyond hemostasis, and exhibit endocytic and phagocytic capabilities potentially related to innate immune mechanisms participating in the rapid removal of pathogens and detecting DAMPs through TLRs [175,176,177]. Alterations of platelet functions have been widely recognized in patients with CKD [170]. A deficient assembly of cytoskeletal proteins was observed in resting and activated platelets from uremic patients [178,179]. These observations, among others, are compatible with biochemical alterations of the platelet contractile system, which may contribute to the impairment of platelet phagocytic and secretory capacities in uremic patients.

Interestingly, platelets are also prime drivers of the inflammatory response occurring at the endothelium. Many interaction pathways convey on the endothelial cell surface linking these two cellular components in the initiation and regulation of hemostasis and inflammation. Inflammation causes the stimulation of both platelets and endothelial cells, affecting not only their role in hemostasis, but also in the immune response [180]. The feedback between endothelial and platelet dysfunctions play a definite role in the alteration of the immune system in patients with uremia.

## 7. Conclusions

CKD is associated with accelerated atherothrombosis, alterations in hemostasis, enhanced inflammatory activity, and an impaired immune response. Development of endothelial damage is widely recognized in CKD patients. The endothelial damage is the result of sustained toxic and inflammatory conditions and contributes to the immune dysfunction developing in these patients.

Alterations in mechanisms of the innate immune system have been reported in patients with end-stage renal disease. Activation of monocytes, macrophages, granulocytes and endothelial cells coexist with a depletion of natural regulatory T-cells and impaired phagocytic functions of polymorphonuclear leukocytes and monocytes. These alterations seem to be aggravated by dialysis procedures.

Endothelial activation is at the cross-road of alterations in inflammatory and immune mechanisms developing in patients with CKD. The feedback between inflammation and immune pathways further potentiates pathologic responses at the endothelial level. A more precise knowledge of the basic molecular mechanisms involved in the development of endothelial damage may facilitate the development of more specific therapeutic strategies that could alleviate the profound alterations in the inflammatory and immunocompetence mechanisms in CKD. Such therapies may in turn reduce the unacceptable death risk from cardiovascular complications in this patient population.

## Abbreviations

**CKD**	Chronic Kidney Disease
**ESRD**	End-stage renal disease
**ED**	Endothelial dysfunction
**ECs**	Endothelial cells
**eGFR**	Estimated glomerular filtration rate
**PAMPs**	Pathogen-associated molecular patterns
**DAMPs**	Damage-associated molecular patterns
**HMGB1**	High-mobility group box 1
**ICAM-1**	Intercellular Adhesion Molecule 1
**VCAM-1**	Vascular Cell Adhesion Molecule 1
**MCP-1**	Monocyte chemoattractant protein-1
**VWF** **ATP**	Von Willebrand Factor Adenosine 5’-triphosphate
**LDL** **NET** **CASP1**	Low density lipoproteins Neutrophil extracellular traps Caspase-1
**IS**	Indoxyl sulfate
**PCS**	p-Cresyl sulfate
**IAA**	Indole-3 acetic acid
**TMAO**	Trimethylamine N-oxide
**ROS**	Reactive oxygen species
**AGEs**	Advanced glycation end-products
**AHR**	Aryl hydrocarbon receptor
**MAPK**	Mitogen-activated protein kinase
**ELAM-1**	Endothelial-leukocyte adhesion molecule
**NFκB**	Nuclear factor kappa B
**TLR4**	Toll-like receptor 4
**NLRP3, NALP3**	NOD-like receptor prying domain-containing-3
**SIRT1**	Sirtuin 1
**KLF2**	Krüppel-like factor 2
**KLF4**	Krüppel-like factor 4
**eNOS**	Endothelial nitric oxide synthase
**VE-cadherin**	Vascular endothelial cadherin
**ZO1**	Zonula occludens
**EPCs**	Endothelial progenitor cells
**MV**	Microvesicles
**EMV**	Endothelial microvesicles
**ECM**	Extracellular matrix
**TF**	Tissue factor
**IL**	Interleukin
**CECs**	Circulating endothelial cells
**PMNs**	Polymorphonuclear leukocytes
**TLRs**	Toll-like receptors
**RTT**	Renal replacement therapy
**PRRs**	Pattern recognition receptors
**ASC**	Apoptosis-associated speck-like protein containing a CARD
**TXNIP**	Thioredoxin-interacting protein
**GSDMD**	Gasdermin D
**VSMCs**	Vascular smooth muscle cells
**MMPs**	Matrix metalloproteinases
**TNF-α**	Tumor necrosis factor alpha
**LPSs**	Lipopolysaccharides
**HD**	Hemodialysis
**HDL**	High-density lipoprotein
**SCFA**	Short-chain fatty acids
**GI**	Gastrointestinal tract
**CV**	Cardiovascular
**PMPs**	Platelet microparticles
